# Gain-of-function variants in *CLCN7* cause hypopigmentation and lysosomal storage disease

**DOI:** 10.1016/j.jbc.2024.107437

**Published:** 2024-06-03

**Authors:** Maya M. Polovitskaya, Tanushka Rana, Kurt Ullrich, Simona Murko, Tatjana Bierhals, Guido Vogt, Tobias Stauber, Christian Kubisch, René Santer, Thomas J. Jentsch

**Affiliations:** 1 Leibniz-Forschungsinstitut für Molekulare Pharmakologie (FMP), Berlin, Germany; 2 Graduate program of Humboldt-Universität zu Berlin and Graduate School of the Max Delbrück Centre for Molecular Medicine (MDC), Berlin, Germany; 3 Department of Pediatrics, University Medical Center Hamburg-Eppendorf (UKE), Hamburg, Germany; 4 Institute of Human Genetics, University Medical Center Hamburg-Eppendorf (UKE), Hamburg, Germany; 5 Institute for Molecular Medicine, Medical School Hamburg (MSH), Hamburg, Germany; 6 NeuroCure Cluster of Excellence, Charité Universitätsmedizin, Berlin, Germany

**Keywords:** chloride transport, electrophysiology, gating, genetic disease, neurological disease, neurodevelopmental disorder, organellar pH homeostasis, patch clamp, vacuolar acidification, organomegaly

## Abstract

Together with its β-subunit OSTM1, ClC-7 performs 2Cl^−^/H^+^ exchange across lysosomal membranes. Pathogenic variants in either gene cause lysosome-related pathologies, including osteopetrosis and lysosomal storage. *CLCN7* variants can cause recessive or dominant disease. Different variants entail different sets of symptoms. Loss of ClC-7 causes osteopetrosis and mostly neuronal lysosomal storage. A recently reported *de novo CLCN7* mutation (p.Tyr715Cys) causes widespread severe lysosome pathology (hypopigmentation, organomegaly, and delayed myelination and development, “HOD syndrome”), but no osteopetrosis. We now describe two additional HOD individuals with the previously described p.Tyr715Cys and a novel p.Lys285Thr mutation, respectively. Both mutations decreased ClC-7 inhibition by PI(3,5)P_2_ and affected residues lining its binding pocket, and shifted voltage-dependent gating to less positive potentials, an effect partially conferred to WT subunits in WT/mutant heteromers. This shift predicts augmented pH gradient-driven Cl^−^ uptake into vesicles. Overexpressing either mutant induced large lysosome-related vacuoles. This effect depended on Cl^−^/H^+^-exchange, as shown using mutants carrying uncoupling mutations. Fibroblasts from the p.Y715C patient also displayed giant vacuoles. This was not observed with p.K285T fibroblasts probably due to residual PI(3,5)P_2_ sensitivity. The gain of function caused by the shifted voltage-dependence of either mutant likely is the main pathogenic factor. Loss of PI(3,5)P_2_ inhibition will further increase current amplitudes, but may not be a general feature of HOD. Overactivity of ClC-7 induces pathologically enlarged vacuoles in many tissues, which is distinct from lysosomal storage observed with the loss of ClC-7 function. Osteopetrosis results from a loss of ClC-7, but osteoclasts remain resilient to increased ClC-7 activity.

Lysosomes are membrane-enclosed organelles that break down various macromolecules and are key regulators of cellular metabolism. For the proper degradation of substrates, lysosomal enzymes need an appropriate ionic environment characterized by acidic pH (1, 2) and high Cl^−^ concentration (3, 4, 5, 6). Luminal acidification of endosomes and lysosomes is carried out by vacuolar-type ATPases. These electrogenic pumps require electric counter currents to prevent the generation of a lumen-positive voltage, which would thereafter inhibit pumping. These currents were conventionally thought to be carried by an influx of chloride ions (7, 8). However, cation efflux is also crucial for lysosomal acidification (9). Endolysosomal CLC 2Cl^−^/H^+^ exchangers, including lysosomal ClC-7, can both electrically shunt H^+^-ATPase currents and accumulate Cl^−^ in the vesicular lumen (5). This renders them essential to the function of endosomes and lysosomes, as evident from genetic diseases (10, 11). As first discovered in mouse models (12, 13), homozygous loss-of-function (LoF) variants in *CLCN7*, the gene encoding ClC-7, and in that encoding its obligatory β-subunit OSTM1 (14), cause infantile malignant autosomal-recessive osteopetrosis (ARO) (MIM: 611490 and 259720). In contrast to a more common form of ARO caused by variants in *TCIRG1* encoding the a3 subunit of the vacuolar-type ATPases (15, 16), *CLCN7*-related osteopetrosis is accompanied by lysosomal storage in neurons and the kidneys (17) and can result in neurodegeneration (15, 18, 19).

Heterozygosity for *CLCN7* variants is found in patients with more benign autosomal-dominant osteopetrosis (ADO) without neurodegeneration (Albers-Schönberg disease, ADO II) (MIM: 166600) (20). Since CLC transporters function as dimers, dominant inheritance may be explained by a dominant-negative effect of the mutant on the WT subunit, or by a deleterious gain-of-function (GoF) of the mutated allele (21, 22). Recently, a novel allelic disorder characterized by hypopigmentation, organomegaly, and delay in axon myelination and development (HOD syndrome, MIM: 618541), was identified in two unrelated patients with the same *de novo* mutation in *CLCN7*. Recapitulated in a mouse model (23), electrophysiological analysis of this mutant (p.Y715C) revealed a marked increase in ion transport activity, indicative of a toxic GoF. Surprisingly, patients carrying this variant lacked osteopetrosis, the hallmark of “classical” *CLCN7* disease. The mechanisms by which the p.Y715C mutant leads to this divergent phenotype remain obscure.

Here, we describe two individuals with symptoms closely resembling those of the previously described HOD syndrome (23). Whereas one of the individuals reported here carries the same p.Tyr715Cys variant, the other is heterozygous for a p.Lys285Thr variant that has not been previously described. Akin to Y715 (24, 25), K285 is located in a suggested binding site for PI(3,5)P_2_ in the cytoplasmic portion of ClC-7, and decreases the response to this lipid, pointing to a common pathogenic mechanism. Similar to p.Y715C, also p.K285T enhances ClC-7 currents and gives rise to enlarged lysosome-like vesicles upon overexpression in mammalian cells. However, p.K285T patient fibroblasts do not display the giant vesicles characteristic of the p.Y715C variant. Despite these minor differences, our work suggests that variants interfering with PI(3,5)P_2_ binding of ClC-7 consistently cause the distinct HOD phenotype. We also propose that altered sensitivity to PI(3,5)P_2_ and PI3P may contribute to ADO with some *CLCN7* variants.

## Results

### *De novo CLCN7* mutations associated with organomegaly, hypopigmentation, and delay in axon myelination and development

**Figure 1:**
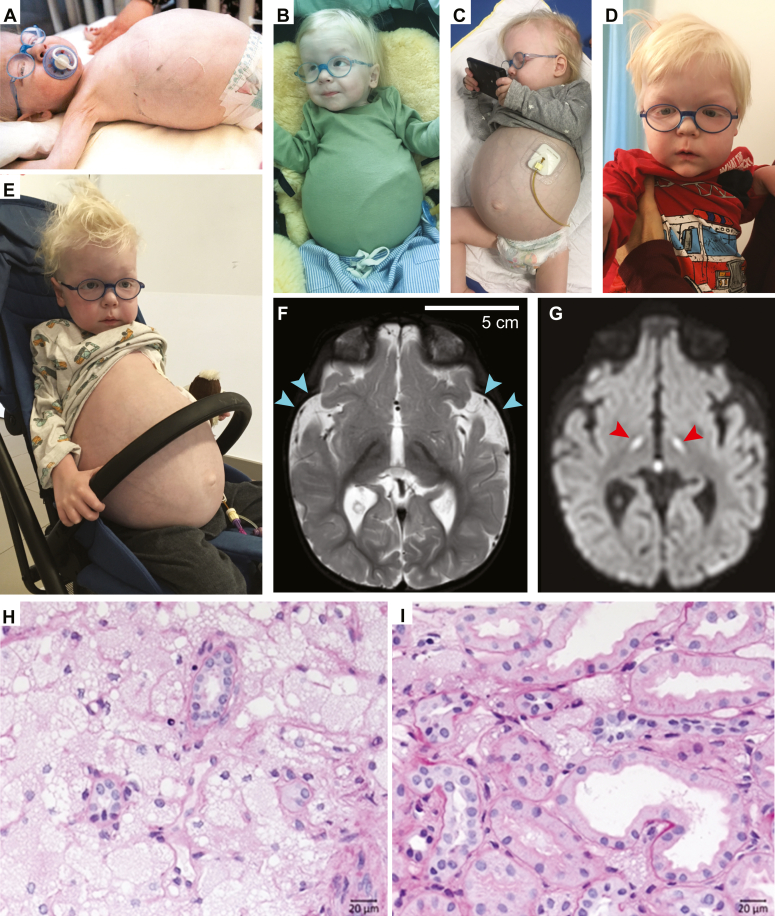
**Clinical, neuroradiographic and histologic features of patient 1.***A*–*E*, patient at ages 18 months (*A* and *B*), 25 months (*C*), 48 months (*D*), and 54 months (*E*). Note hypopigmentation of hair including eyebrows but not the irides, the massively distended abdomen due to organomegaly, and a gastric tube in place because of impaired intestinal transit. *F* and *G*, T2-weighted (*F*) and diffusion-weighted (*G*) cranial MRI scans at age 14 months showing frontotemporally accentuated brain volume reduction (*cyan arrowheads*), mildly delayed myelination and symmetrical signal alterations in the nucleus subthalamicus (*red arrowheads*), as well as in the medulla oblongata and the cerebellar peduncles (not shown) (*G*). *H* and *I*, periodic acid-Schiff (PAS) staining of kidney biopsies obtained at 19 months of age. The large unstained circular regions are most likely intracytoplasmic vacuoles, although lipid droplets cannot be rigorously excluded.

We report two unrelated males with hypopigmentation, muscular hypotonia, failure to thrive, organomegaly, delayed myelination, and psychomotor developmental disorder. Patient 1 (Fig. 1, *A*–*E*) was referred to diagnostic trio exome analysis that revealed heterozygosity for a *de novo CLCN7* variant, c.854A>C [p.(Lys285Thr)], as the most probable genetic cause of disease (Fig. S1). This variant is absent in the gnomAD database (v.4.0) and is predicted to be deleterious by various commonly applied bioinformatics prediction programs with a CADD score of 28.6 and a REVEL score of 0.872. This finding, and the publication of the first two HOD cases (23), prompted the targeted Sanger sequencing of the *CLCN7* gene in the preserved fibroblast sample of a similar clinical case (patient 2), who passed away in 1999 without genetic diagnosis. As a result, the previously described (23) p.Tyr715Cys variant was detected in a heterozygous state (Fig. S1).

Patient 1 (aged 6 years and 4 months at the time of this report) presented at around 8 months of age with hypopigmentation, strabismus, muscular hypotonia, and a primary psychomotor developmental disorder. A cranial MRI at 14 months of age revealed delayed myelination in addition to reduced cerebral volume, particularly evident in the frontotemporal region. Furthermore, the patient had enlarged and degenerated olivary bodies with symmetrical hyperintensities of the subthalamic nucleus (Fig. 1, *F* and *G*). Over time, he also developed grossly enlarged organs with fully preserved function (liver, spleen, and kidneys). A liver biopsy taken at 19 months of age revealed numerous CD68^+^ histiocytic cells, particularly in the portal fields, with foamy, highly expanded, and light-colored cytoplasm. Similarly, a kidney biopsy showed numerous macrophages transformed into foam cells mainly in the medullary interstitium (Fig. 1, *H* and *I*). Leukocytes also showed severe vacuolization. A mild thickening of the left ventricular myocardium was first observed at 5 years of age (posterior wall of left ventricle: 9.0 mm; reference range: 5.2 ± 1.7 mm). X-ray examinations of the skeleton at 6 years showed significantly delayed bone age (3 years), but no structural anomalies. The patient’s plasma chitotriosidase, a biomarker representing tissue-resident macrophage activation upon excessive lipid accumulation, was detected to be tremendously elevated (up to 18,400 nmol/h/ml; reference range <150 nmol/h/ml). Other biomarkers of lysosomal function and numerous enzymatic studies yielded essentially normal results (Table S1). A markedly elevated concentration of bound neuraminic acid suggestive of neuraminidase deficiency was only returned after results of genetic testing.

The patient’s clinical course is progressive despite treatment with chloroquine, a drug known to increase lysosomal pH, and promising results in a *Fig4* null mouse model that phenocopies HOD syndrome (26), as well as in the previously described (23) p.Y715C patients (E. R. Nicoli, D.-L. Day-Salvatore, personal communication). This treatment was started at the age of 19 months with the aim to achieve a blood concentration of 150 to 200 μg/l (as suggested and well-tolerated in malaria treatment). There has been no marked regression in neurological development.

The clinical picture is dominated by progressive thickening of bowel walls, resulting in chronic impairment of intestinal transit, an extremely distended abdomen, malabsorption, loss of protein and electrolytes, and failure to thrive with length at the time of this report −2.93z (2.93 standard deviations below the mean), weight −1.47z, head circumference −0.97z compared to normal values at this age. This, together with recurrent enterogenic infections and aspirations, led to the need for tracheostomy and ventilation.

Patient 2 (deceased 1999, aged 15 months) was referred at the age of 6 months because of recurrent diarrhea, proteinuria, failure to thrive, impaired psychomotor development, microcephaly, hepatosplenomegaly, muscular hypotonia, and partial albinism. Anthropometric measurements at the time recorded length as −3.3z, weight −4.0z, and head circumference −5.9z. A lysosomal storage disorder was considered due to immense vacuolization observed in lymphocytes, polymorphonuclear cells, in biopsy material of the enlarged kidneys, and the detection of foam cells in a bone marrow aspirate. A neuraminidase deficiency was initially considered because repeated measurements in different laboratories had shown decreased enzyme activity in fibroblasts with increased bound neuraminic acid in these cells (Table S1). This was ultimately interpreted as secondary to an unknown underlying metabolic disorder, since the decrease in enzyme activity was rather mild and accompanied by a normal K_m_ value, and oligosaccharides and neuraminic acid in urine were reported normal. Further, sequencing of the *NEU1* gene revealed only WT sequence. Routine X-ray examinations of the skull and left hand were age-appropriate, without evidence of a mineralization disorder. The patient developed increasing nutritional problems, diarrhea, and edema, as well as an *Escherichia coli* septicemia before he died. A postmortem examination confirmed the myelination disorder already known from a cranial MRI and showed perivascular accumulation of storage macrophages and vacuolization of neuronal cells within the relatively small brain (580 g, average for age 944 g).

To explore the effects of the novel *CLCN7* variant p.K285T and compare them with those of the previously published p.Y715C, we performed patch-clamp electrophysiology and investigated lysosomal morphology and function *in vitro*.

### Effects of *CLCN7* variants on ClC-7/OSTM1 ion transport

For electrophysiological analyses of ClC-7 2Cl^−^/H^+^ exchange currents, we targeted human ClC-7 and its disease-associated mutants to the plasma membrane using the ClC-7^LL23/24AA,LL68/69AA^ mutant (referred to as WT in this section), in which lysosome-targeting dileucine motifs have been eliminated (27). Cells were cotransfected with complementary DNA encoding the obligatory ClC-7 β-subunit OSTM1 (14) carrying a C-terminal GFP label to identify transfected cells. In order to characterize responses to acidic extracellular pH (pH_o_) that mimics the luminal pH of lysosomes, we used *TMEM206*^−/−^ HeLa cells (28). They lack acid-sensitive outwardly rectifying (ASOR) anion channel which is the main, ubiquitously expressed acid-activated Cl^−^ channel. As acid-sensing cation channels, ASICs, rapidly inactivate, *TMEM206*^−/−^ cells provide a virtually background-free system to study transport activity of ClC-7 at acidic pH.

**Figure 2:**
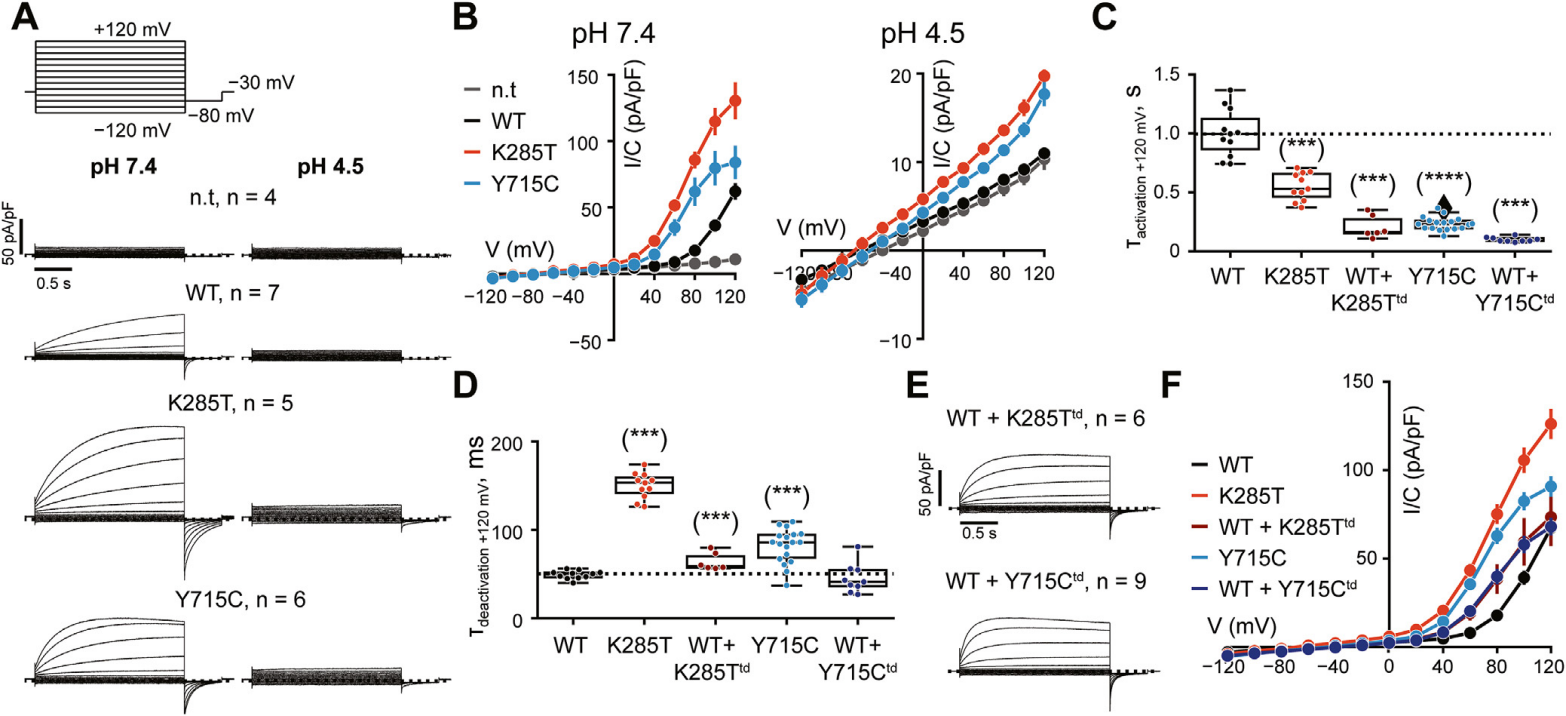
**Electrophysiological properties of ClC-7**^**K285T**^**.***A*, averaged whole-cell current traces recorded from nontransfected (n.t.) HeLa *TMEM206*^−/−^ cells, and cells transfected with plasma membrane-targeted variants of ClC-7/OSTM1-GFP not carrying additional mutations (WT), ClC-7^K285T^ (K285T), ClC-7^Y715C^ (Y715C) in response to the voltage clamping protocol shown above. Measurements were recorded sequentially and on the same cell at extracellular pH 7.4 (*left*) and 4.5 (*right*). Number of measurements is indicated as n. *B*, current-voltage relationship of the currents at pH 7.4 (*left*) and pH 4.5 (*right*) shown in (*A*). Note, that current densities of K285T and Y715C are likely underestimated due to the toxicity of high expression levels, leading to a bias of measured cells. *C*, time constant of activation obtained from single exponential fits of traces at +120 mV at bath pH 7.4 of the conditions described above as well as from cells coexpressing WT ClC-7 and ClC-7^K285T,E314A^ (K285T^td^) or ClC-7^Y715C,E314A^ (Y715C^td^) together with OSTM1-GFP to mimic heterozygosity. *D*, time constant of deactivation obtained from single exponential fits of tail currents at −80 mV after the 2-s +120 mV voltage step at bath pH 7.4. *E*, averaged whole-cell current traces recorded as in (*A*). *F*, current-voltage relationship of the currents at pH 7.4 of WT ClC-7, ClC-7 carrying the patient mutations, and coexpression of WT ClC-7 with ClC-7^K285T,E314A^ (K285T^td^) or ClC-7^Y715C,E314A^ (Y715C^td^) at 1:1:1 mutant:WT:OSTM1 DNA ratio. In (*C*, *D*, and *F*), measurements from (*A*) and (*B*) are pooled together with additional ones, totaling n = 11 for WT and K285T, and n = 18 for Y715C. In (*B* and *F*), error bars represent standard error of the mean (SEM). *Boxes* indicate the median and quartiles of the distribution. ∗∗∗, *p* < 0.001, ∗∗∗∗, *p* < 0.0001 (against WT, Mann-Whitney U test; false-discovery rate was controlled using the Benjamini–Hochberg procedure).

As described previously (21), WT ClC-7 exhibited slowly activating and strongly outwardly rectifying currents at pH_o_ 7.4 in whole-cell patch-clamp experiments. These currents were absent from nontransfected cells (Fig. 2, *A* and *B*). K285T, analogous to Y715C (23), showed markedly increased current amplitudes at cytoplasmic-positive potentials. Their voltage dependencies appeared shifted toward less positive cytoplasmic potentials (Fig. 2, *A* and *B*).

Surprisingly, lowering pH_o_ to 4.5, which mimics the native luminal environment of lysosomes, suppressed WT ClC-7 currents to background levels (Fig. 2, *A* and *B*). Currents of both disease variants, K285T and Y715C, while retaining sensitivity to acidic pH, could still be detected above background.

Activation kinetics upon steps at +120 mV was assessed by fitting current traces with single exponential functions. Activation was faster with both K285T and Y715C variants than in WT (Fig. 2*C*). By contrast, current deactivation, obtained from tail currents at −80 mV after a 2-s voltage pulse to +120 mV, was slower in the disease variants (Fig. 2*D*). Larger current density at both neutral and acidic pH, faster activation and slower deactivation may be multiple facets of the GoF phenotype.

In all patients known to date, HOD-causing mutations are present in a heterozygous state, in which mutant/mutant ClC-7 dimers are predicted to constitute only one-fourth of the total number of transporters. Although a strong GoF even in a minority of transporters can be detrimental, we investigated how mutations affect the function of heteromeric WT/mutant transporters. To this end, we coexpressed WT ClC-7 with doubly mutated ClC-7^K285T,E314A^ or ClC-7^Y715C,E314A^, in which the patients’ mutations were combined with the “proton” glutamate mutation that almost completely abolishes ClC-7 current (21, 29). In this experimental setting, current can only flow through the WT subunits and changes in voltage dependence and activation kinetics reflect the influence of the mutated subunit on the common gate acting on both subunits (22). When WT and doubly mutated ClC-7 were coexpressed at 1:1 DNA ratio, the resulting current exhibited altered voltage dependence of gating that is consistent with the strong dominant effect of the patient mutation with a contribution of the WT/WT homodimers (Fig. 2, *E* and *F*). Strikingly, their activation kinetics were even faster than those of the corresponding “homozygous” K285T and Y715C homodimers (Fig. 2, *A*, *C* and *E*).

### ClC-7^K285T^ lost sensitivity to inhibition by PI(3,5)P_2_

**Figure 3:**
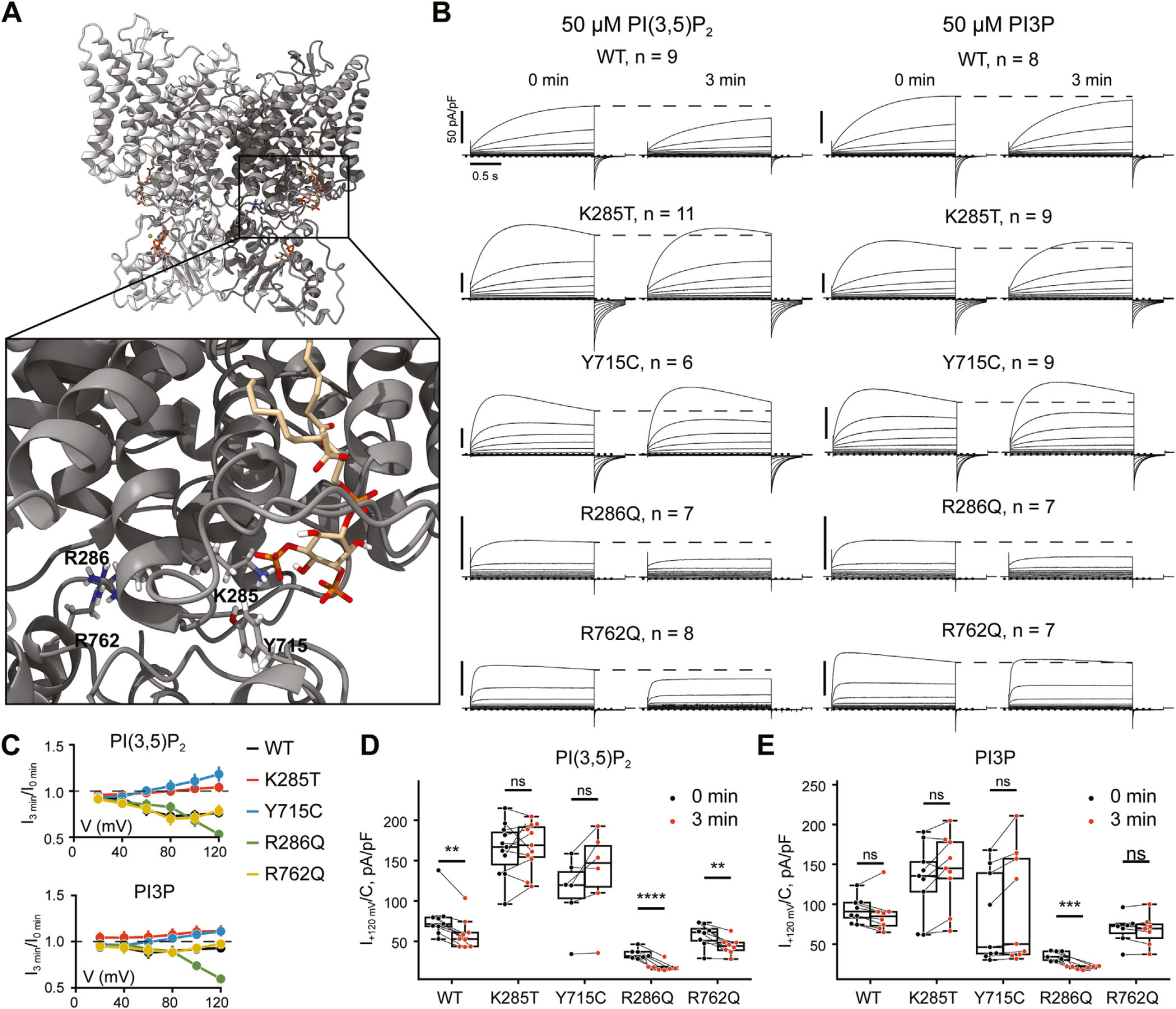
**K285T, like Y715C, is insensitive to diC8-PI(3,5)P**_**2**_**.***A*, 3D structure of ClC-7 dimer with bound PI(3,5)P_2_ (*beige*). Residues under investigation are shown in *stick* representation. PDB: 7JM7 (25). *B*, averaged whole-cell current traces recorded from HeLa *TMEM206*^−/−^ cells transfected with the specified ClC-7 variants, immediately after rupturing the membrane at 0 min and 3 min later, when diC8-PI(3,5)P_2_ had diffused into the cell. *Dotted line* indicates the pseudosteady state current at 0 min that was used for the analysis. Number of cells is indicated as n, measurements were obtained over at least two independent transfections. *C*, ratio between the current amplitude after 3 min of cytosol dialysis and the initial current amplitude of the currents shown in (*B*). Error bars represent SEM. *D* and *E*, current densities at +120 mV of the measurements averaged in (*B*). *Boxes* indicate the median and quartiles of the distribution. ∗∗*p* < 0.01; ∗∗∗*p* < 0.001; ∗∗∗∗*p* < 0.0001; ns, not significant (paired *t* test; false-discovery rate controlled using the Benjamini–Hochberg procedure). PDB, Protein Data Bank.

ClC-7 is tonically inhibited by PI(3,5)P_2_—an inhibition which is lost when Y715 is mutated in previously reported HOD patients (24). Although separated in the primary amino acid sequence of ClC-7, both Y715 and K285 are located in the spatial vicinity of the phosphoinositide head group (Fig. 3*A*), suggesting these mutants share a pathogenic mechanism. Indeed, the addition of 50 μM diC8-PI(3,5)P_2_, but not of diC8-PI3P, to the patch pipette solution mildly yet consistently inhibited WT, but not ClC-7^K285T^ or ClC‑7^Y715C^ transporter currents (Figs. 3, *B*–*D* and S2).

Intriguingly, a few previously known osteopetrosis-causing variants (30, 31, 32) are also located in the vicinity of the polyphosphoinositide binding site (25). Some of them accelerate gating and for this reason they were tentatively interpreted as GoF variants (33). To investigate whether the loss of PI(3,5)P_2_ sensitivity differentiates the known HOD-causing mutations (24) from ADO-causing mutations with accelerated gating, we chose R286Q, a mutation in a residue adjacent to K285 (30). In contrast to K285, the side chain of R286 is turned away from the inositol head group and is therefore unlikely to participate directly in its binding (Fig. 3*A*). Similar to K285T and Y715C, R286Q showed accelerated gating kinetics, but unlike K285T and Y715C, R286Q did not shift the voltage dependency to less positive potentials (21). Unexpectedly, R286Q exhibited stronger inhibition by diC8-PI(3,5)P_2_ than WT ClC-7 (Figs. 3, *B*–*D* and S2). Unlike WT, R286Q could also be suppressed by diC8-PI3P (Figs. 3, *B*, *C* and *E* and S2). We hypothesized that increased sensitivity to lysosomal polyphosphoinositides may result in stronger tonic inhibition of R286Q in the native lysosomal membrane compared to WT, thereby causing a LoF phenotype. That would render its faster activation by voltage irrelevant for pathogenesis. To test this idea, we investigated R762Q, another fast-gated mutation found in ADO (34) and in a compound heterozygous state in ARO (12). This residue is located in the cystathionine beta-synthase 2 domain and faces R286. The response of R762Q to diC8-PI(3,5)P_2_ or diC8-PI3P was indistinguishable from WT (Figs. 3, *B*–*E* and S2). Hence, a loss of PI(3,5)P_2_ sensitivity is characteristic of the HOD-causing *CLCN7* mutations known till date, and increased sensitivity to polyphosphoinositides is observed in some, but not all, osteopetrosis-associated accelerated gating mutants. It does not constitute a general pathogenic mechanism of ADO.

### ClC-7^K285T^ leads to giant vesicles in transfected cells and mildly enlarged lysosomes in subject’s skin fibroblasts

**Figure 4:**
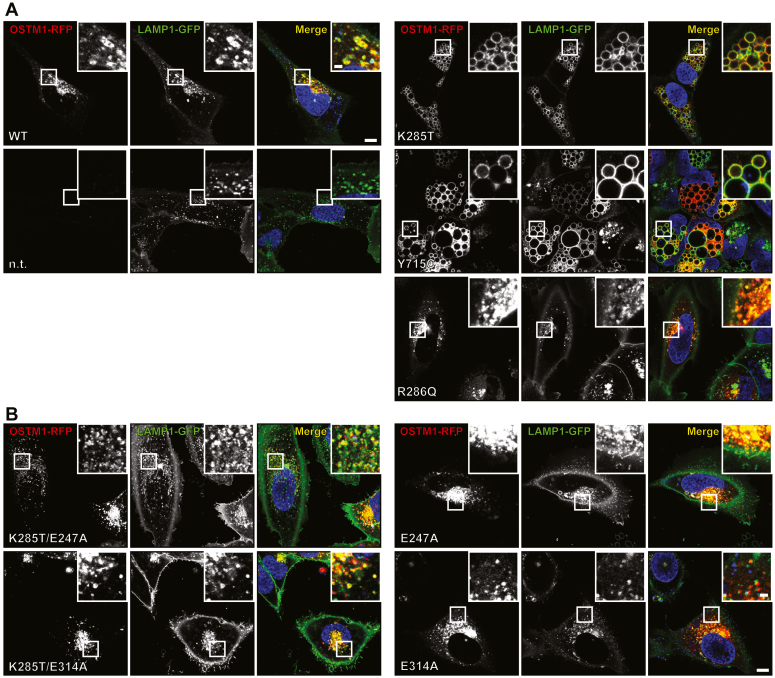
**Effect of overexpression of ClC-7 mutants on LAMP1-positive vesicles.***A*, live cell imaging of U2OS cells constitutively expressing LAMP1-GFP overexpressing ClC-7-P2A-OSTM1-RFP, carrying the HOD-causing patient mutations (Y715C and K285T), ADO-causing “fast” mutation (R286Q), as well as nontransfected cells (n.t.). *B*, live cell imaging of U2OS cells overexpressing and expressing uncoupling mutation (E247A), transport-deficient (E314A), as well as double mutants carrying the patient mutation together with one of the two latter mutations (K285T/E247A and K285T/E314A). Vesicle enlargement caused by K285T overexpression can be suppressed by either uncoupling (E247A mutant) or impairing (E314A mutant) the ion transport by ClC-7. The scale bars represent 10 μm in the overview panels and 2 μm in the magnified panels. ADO, autosomal-dominant osteopetrosis; HOD, hypopigmentation, organomegaly, and delay in axon myelination and development. RFP, red fluorescent protein.

We coexpressed WT or K285T mutant ClC-7 with OSTM1-red fluorescent protein (RFP) in an osteosarcoma U2OS cell line that constitutively expressed LAMP1-GFP (35) to assess the influence of the K285T variant on lysosome morphology. Overexpression of either ClC-7^K285T^ or ClC-7^Y715C^ (24), but not WT ClC-7 or ADO-causing ClC-7^R286Q^, resulted in the generation of strikingly large LAMP1-GFP positive vesicles (Figs. 4*A*, S3*A*). Similar huge vesicles were also recently described upon transfection of rat ClC-7^Y713F^, the murine equivalent of the same tyrosine residue affected in the first HOD patients (36). The generation of these giant vesicles depended on the Cl^−^/H^+^ exchange activity of ClC-7. Combining K285T with the uncoupling mutation E247A, which turns the ClC-7 exchanger into a nongated Cl^−^ channel (21), abolished vesicular enlargement (Figs. 4*B* and S3*A*). Mutating the “proton glutamate” E314A, which almost completely suppresses current (21, 29), likewise prevented vesicle enlargement by K285T (Figs. 4*B* and S3*A*).

**Figure 5:**
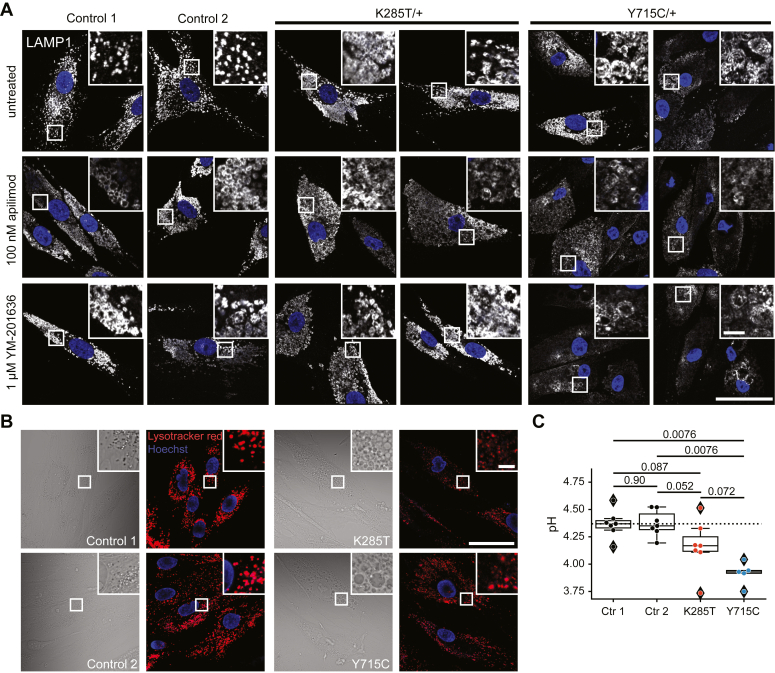
**Lysosomal morphology and pH in subjects’ dermal fibroblasts.***A*, late-endosomal and lysosomal marker LAMP1 in control and subject fibroblasts, either untreated (*top row*) or upon PIKfyve inhibition by apilimod (100 nM) (*middle row*) or YM-201636 (1 μM) (*bottom row*). The scale bars represent 50 μm in the overview panel and 5 μm in the magnified panel. *B*, live-cell images of subjects’ fibroblasts stained with LysoTracker Red DND-99 (*red*). Nuclei were visualized with Hoechst 33342 (*blue*). Phase contrast images (*left*) show moderately enlarged lysosomes and robustly enlarged vacuoles in K285T and Y715C fibroblasts, respectively. The scale bars represent 50 μm in the overview panel and 5 μm in the magnified panel. *C*, lysosomal pH values in fibroblasts from subjects 1 and 2, and from two healthy controls obtained from OG488-Dextran fluorescence ratio at 436 and 480 nm. *Circles* represent mean values obtained from two technical replicates (dishes) each having at least four fields of view imaged. *Boxes* indicate the median and quartiles of the distribution. Exact *p*-values, corrected for multiple comparisons, are stated over the horizontal lines connecting the corresponding pairs of conditions (Mann–Whitney U test; false-discovery rate controlled using the Benjamini–Hochberg procedure).

While kidney biopsies of patient 1 with the K285T mutation showed histiocytic vacuolization, LAMP1-positive compartments in cultured skin fibroblasts were not drastically enlarged. This lack of enormous vesicles stood in contrast with fibroblasts from subjects carrying the Y715C mutation (Figs. 5*A*, S3*B*) (23). However, in comparison with control fibroblasts, where LAMP1-positive compartments appeared as punctae, *CLCN7*^K285T/+^ fibroblasts contained many vesicles with a clearly visible lumen, suggesting that they were mildly enlarged (Figs. 5*A* and S3*B*). A similar picture was seen in live cell imaging experiments, which showed moderately enlarged vesicles in *CLCN7*^K285T/+^ fibroblasts (Fig. 5*B*).

The discrepancy in vesicle size between *CLCN7*^K285T/+^ and *CLCN7*^Y715C/+^ cells prompted us to test whether vesicles of *CLCN7*^K285T/+^ fibroblasts retained residual sensitivity to inhibition by PI(3,5)P_2_, that could not be detected in our patch-clamp experiments (Fig. 3). Lysosomal PI(3,5)P_2_ originates solely from PI3P phosphorylation by PIKfyve (37). We applied inhibitors of PIKfyve, which are known to cause lysosomal enlargement (38). Apilimod (100 nM) or YM-201636 (1 μM) treatment for 3 h resulted in large LAMP1-positive compartments in both control fibroblast lines, but had no effect on giant vesicles of *CLCN7*^Y715C/+^ cells. Upon PIKfyke inhibition, LAMP1-positive compartments of *CLCN7*^K285T/+^ cells from patient 1 approached the size of those seen in *CLCN7*^Y715C/+^ cells (Figs. 5*A*, S3*B*). This observation suggests that ClC-7^K285T^ may have dampened, but not completely abolished sensitivity to PI(3,5)P_2_.

### Lysosomal pH in K285T and Y715C patient fibroblasts

Ratiometric measurements of lysosomal pH in *CLCN7*^Y715C/+^ fibroblasts from the first two reported subjects revealed luminal hyperacidification (23). We investigated fibroblasts obtained from skin biopsies of the newly described patients using Oregon Green (OG) 488-coupled Dextran, and compared them with two cell lines obtained from healthy controls. Control lines yielded lysosomal pH values of ∼4.36 (Fig. 5*C*). Both *CLCN7*^K285T/+^ and *CLCN7*^Y715C/+^ patients fibroblast lines showed a tendency toward luminal pH values that were lower than controls by ∼0.2 and 0.4 pH units, respectively (Fig. 5*C*). Further, the measured luminal pH in *CLCN7*^Y715C/+^ fibroblasts was consistent with the previous report (23). These values may be an underestimation, because the more diffusely stained larger vesicles in *CLCN7*^Y715C/+^ fibroblasts could not be individually sampled by our automated detection protocol (see Lysosomal pH Measurement in the Experimental procedures section). Moreover, impaired acidification in the larger vacuoles may also be inferred from the lack of LysoTracker Red DND-99 labeling, which is detected upon entrapment in acidic compartments with fluorescence following protonation (Fig. 5*B*).

## Discussion

Variants in *CLCN7*, which encodes the lysosomal 2Cl^−^/H^+^ exchanger ClC-7, lead to a puzzling spectrum of lysosome-related pathologies in humans. *CLCN7* LoF mutations entail osteopetrosis and, dependent on the degree of LoF, seemingly neuron- and kidney-specific lysosomal storage and neurodegeneration. Comparatively milder osteopetrosis is also observed in patients heterozygous for gating-accelerating mutations that were tentatively classified as causing a GoF. A particular GoF mutation, Y715C, has recently been shown to cause HOD, a different lysosome-related syndrome. Patients, as well as mice engineered to carry the corresponding variant, surprisingly lack osteopetrosis while displaying albinism and severe lysosomal pathology in many organs. The relationship between altered ion transport properties of these ClC-7 variants and clinical symptoms have remained unclear.

Here, we have identified another individual (patient 2) with typical HOD symptoms and the previously described Y715C mutation, strengthening the genotype–phenotype relationship. Importantly, we also identified an individual (patient 1) with a different *de novo* mutation (K285T), presenting with a similar spectrum of symptoms, and have characterized the effect of this mutation on the transport properties of ClC-7 and lysosome morphology and function.

### Changes in ClC-7 transport properties and regulation by HOD-causing ClC-7 mutants

Both the Y715C and K285T mutations had very similar effects on ClC-7 currents—an apparent shift in the voltage-dependence of gating to less positive cytoplasmic voltages, as well as changes in gating kinetics. The side chains of K285 and Y715 lie in the vicinity of a phosphoinositide identified in cryo-EM studies (25). Patch-clamp experiments of plasma membrane-targeted ClC-7/OSTM1 revealed that both mutants had lost sensitivity to PI(3,5)P_2_. This suggests that both residues contribute to the binding of PI(3,5)P_2_, a lysosomal phosphoinositide that suppresses ClC-7 transport (24). Because the shift of voltage-dependence was observed at the plasma membrane which lacks PI(3,5)P_2_, it likely results from a conformational change of the protein that is independent of the loss of PI(3,5)P_2_ binding. This conclusion is supported by our experiments directly testing the effect of PI(3,5)P_2_ on ClC-7 currents (Figs. 3 and S2). It is unclear whether the impaired regulation of ClC-7 by PI(3,5)P_2_ is necessary or sufficient to cause HOD, or whether the observed changes in voltage-dependence *per se* suffice to cause this pathology. The latter notion is supported by the fact that pathogenic *CLCN6* mutations that similarly shift the voltage-dependence of ClC-6 to more negative voltages affect residues that cannot be implicated in binding PI(3,5)P_2_ (39, 40). Of note, decreased inhibition by PI(3,5)P_2_ would further increase ClC-7 currents.

Changes in voltage-dependence may have dominant effects, even if affecting only mutant, but not WT ClC-7 subunits. However, like all CLCs, ClC-7 functions as a dimer. In WT/mutant heteromers, predicted to represent 50% of total ClC-7 in heterozygous patients, the voltage-dependence of a WT subunit may be influenced by the attached mutant subunit. In fact, many dominant myotonia-causing ClC-1 mutations shift the voltage-dependence of attached WT subunits to positive potentials, causing a dominant LoF (41, 42). Akin to common gating in ClC-0 and ClC-1 (10), ClC-7 gating involves both subunits of the dimer (22). This implies that both subunits of WT/mutant heteromers gate with the same voltage-dependence and kinetics. ADO mutants partially confer their accelerated gating to WT subunits in ClC-7 heteromers (22). Here, we showed that ion-transport-deficient ClC-7^K285T,E314A^ and ClC-7^Y715C,E314A^ double mutants altered the voltage dependence of attached WT subunits (Fig. 2, *E* and *F*). Hence, depending on the magnitude of the voltage-shift in WT/mutant heteromers, the effect on cellular processes and disease outcome may be greater than expected for a superposition of WT and mutant currents.

### Effect on size and pH of lysosome-like vesicles

Both ClC-7^Y715C^ and ClC-7^K285T^ generated large LAMP1-positive vesicles upon overexpression in transfected cells. Mutagenesis showed this effect was contingent on their Cl^−^/H^+^ exchange activity, in line with previous reports for WT ClC-3 (43), ClC-6^Y553C^ and ClC-6^T520A^ (39, 40), and ClC-7^Y715C^ (23, 36). This observation suggests that overactive ClC-7 would increase pH-gradient driven luminal Cl^−^ uptake. This notion is also supported by the normalization of lysosome-size in FIG4-deficient cells by the pH gradient-disrupting drug chloroquine (26) or by disruption of *CLCN7* (44). The resulting increased luminal osmotic pressure would then inhibit vesicle fission, but not fusion, thereby resulting in the generation of large vacuoles (39).

While both mutants generated large vesicles upon overexpression, giant vacuoles were observed only in fibroblasts from subjects carrying the Y715C mutation. This disparity may be owed to a less pronounced loss of PI(3,5)P_2_ sensitivity with ClC-7^K285T^. Indeed, the enlargement of ClC-7^K285T^, but not ClC-7^Y715C^ lysosomes by PIKfyve inhibition suggests that ClC-7^K285T^ retains some PI(3,5)P_2_ sensitivity, although this was not observed in our patch-clamp experiments. Of note, however, for technical reasons these experiments used diC8-PI(3,5)P_2_, a shorter fatty acid chained phosphoinositide. The lower biological efficacy of diC8 compared to natural polyphosphoinositides has been previously observed for various target proteins (45, 46).

Lysosomes of fibroblasts from both patients showed tendency toward more acidic values than their WT counterparts, with only the difference between WT and p.Y715C being statistically significant.

Whereas steady-state lysosomal pH does not depend on ClC-7 (5, 14, 17, 24), ClC-7 fosters acidification when PI(3,5)P_2_ inhibition is relieved pharmacologically or by the Y715C mutation (24). The apparent differences in pH, however, must be interpreted with caution due to inherent difficulties in measuring pH_lys_, and to differences in vesicle sizes. Differences in pH_lys_ may underlie varying effectiveness of treatment with chloroquine, a pH gradient-dissipating drug. Whereas partial remission of symptoms in p.Y715C patients (23) by chloroquine treatment was reported, disease progression could not clearly be slowed down, nor did it alleviate symptoms in our patient 1. These observations however, remain anecdotal and require larger cohorts for validation.

As reported previously for p.Y715C (23), an increase in acidification was only observed in lysosome-like vesicles of near-normal size. The drastically enlarged vacuoles of p.Y715C fibroblasts were poorly acidified. This weak acidification was not an artifact of poor endocytic uptake of OG 488-coupled Dextran since these vacuoles also stained poorly for LysoTracker Red DND-99. Poor acidification of large vacuoles does not contradict the notion that H^+^-gradient driven Cl^−^ uptake underlies their generation. Equilibrium calculations for 2Cl^−^/H^+^-exchange predict that even a luminal pH of 6.8 would support a >140 mM/30 mM ratio of [Cl^−^]_lum_/[Cl^−^]_cyt_ with cytoplasmic pH = 7.2 and lumen-negative V_lum_ = −20 mV. The cause of the poor acidification remains to be clarified.

### Lysosomal voltage, a parameter crucial for vesicular CLCs

The pathological consequences of the observed shift in ClC-7 voltage-dependence identify lysosomal voltage as a crucial parameter. While there are reasonable estimates for lysosomal concentrations of H^+^ (pH), Cl^−^, Na^+^, K^+^, and Ca^2+^, estimates for the voltage of lysosomes and related compartments vary widely depending on the method used. Values between −40 mV and +40 mV (V_lum_ – V_cyt_) have been obtained thus far (47, 48). A recently reported strongly lumen-positive lysosome potential (V_lum_ ≈ +114 mV) (49) is less likely. Such a potential would not allow the efficient operation of numerous lysosomal ion transporters, most of which require lumen-negative voltages for activity. Given the ion gradients over lysosome membranes, such potential cannot be generated by ionic diffusion potentials. Traditionally, lysosomes were assumed to be inside-positive due to the active transport of positive charge into these compartments by H^+^-ATPase (1). This, however, ignores diffusion potentials generated by channels or electrogenic transporters. Reductionist (5) and subsequent more elaborate (50, 51) model calculations showed that parallel operation of a 2Cl^−^/H^+^ exchanger and an H^+^-ATPase should rather lead to moderately inside-negative voltages (V_lum_ ≈ −20 to −40 mV). Likewise, the parallel operation of so-called “two-pore channel” (TPC) cation channels and ASOR/TMEM206 anion channels predicts V_lum_ ≈ −20 mV for macropinosomes (52), a value largely determined by the strong outward rectification of ASOR. Importantly, these moderately negative luminal potentials are compatible with the apparent threshold of activation of all vesicular CLCs (21, 53) with the exception of ClC-6 (54). Intriguingly, the vesicular anion channels ASOR/TMEM206 (28, 52, 55, 56) and CLN7 (57), as well as cation channels two-pore channel 1 (58), TRPML1, and TRPML2 (59, 60) display steep activation by voltages beginning at V_lum_ ≈ −20 to −30 mV. Such endolysosomal voltages would allow regulation of vesicular ion transport in voltage-mediated feedback loops (52). We take the clear effects of voltage-dependence shifting ClC-7 and ClC-6 mutants on vesicle size as an additional, strong indication for physiological, steady state V_lum_ being around −20 to −30 mV.

As an exception, ClC-6 strongly activates at V_lum_ <≈ −100 to −120 mV (54). We deem it highly unlikely that this value reflects physiological, steady state values of V_lum_. It seems feasible, however, that such voltages might be achieved transiently. ClC-6 also gives small currents at −20 to −40 mV (61), and model calculations show that even small currents below detection limits may suffice to accumulate Cl^−^ into lysosomes (Supplementary Note).

### Acceleration of gating as a possible pathogenic factor

Assuming lysosomal steady state voltage is roughly −20 to −30 mV, the altered voltage dependence of ClC-7 leads to a strong gain of function by increasing currents at physiological potentials. In addition, both Y715C and K285T mutations accelerated the voltage-gating of ClC-7. This is reminiscent of several dominant osteopetrosis-causing *CLCN7* mutations, for instance R286Q and R762Q (21, 22, 34). In contrast to the HOD-associated K285T and Y715C mutants, which show faster voltage-dependent activation and slower deactivation, the ADO II mutants accelerated both activation and deactivation. This physiological distinction may be pathologically relevant. With short excursions to more lumen-negative lysosomal voltages, as may occur during transient opening of lysosomal Ca^2+^- or Na^+^-channels (58), faster activation, together with slower inactivation, may further increase ClC-7 currents of HOD-mutants after returning to steady-state voltages. This will not be the case with R286Q and R762Q ADO II-causing mutants (which importantly lack the shift in voltage-dependence).

The function of the slow voltage-dependent activation of both ClC-7 (21) and ClC-6 (54), which is not observed in ClC-3, ClC-4, or ClC-5, remains unclear. It may delay repolarization of Na^+^- or Ca^2+^-release-mediated voltage excursions of lysosomes (58), acting similar to delayed rectifier K^+^ channels in neuronal action potentials. Acceleration of ClC-7 gating may represent a toxic GoF that blunts lysosomal voltage excursions and thereby enhances the efflux of Na^+^ or Ca^2+^ by diminishing the negative feedback provided by voltage. However, it is unclear why accelerated gating in ADO II-linked mutants is associated with dominant osteopetrosis, which is also observed with a loss of ClC-7 function, whereas osteopetrosis is not observed with the HOD-associated mutations K285T and Y715C mutants.

### Genotype-phenotype relationship of *CLCN7* variants

ClC-7 being a lysosomal protein, variants in *CLCN7* result in lysosome-related pathologies. These include lysosomal storage, osteopetrosis, and pigmentation defects (33). ClC-7-related osteopetrosis is owed to the impaired formation and/or function of the acid-secreting ruffled border of osteoclasts that is created by lysosome exocytosis (12). Pigmentation defects may be explained by dysfunction of melanosomes as in other lysosomal diseases (62, 63, 64).

Unambiguous loss of ClC-7 function results in osteopetrosis, lysosomal storage in the brain and kidneys, as well as pigmentation defects in mice (12, 21, 33). By contrast, a clear gain of ClC-7 function, exemplified by the activity-enhancing mutations analyzed here, does not cause osteopetrosis, but lysosomal pathology and pigmentation defects, which differ from those observed by a loss of ClC-7. It appears that lysosomal exocytosis, which is essential for the function of osteoclasts, requires ClC-7 activity (5, 12, 65). ClC-7 activity can be increased further without detrimental effects on osteoclasts. The mechanisms underlying lysosomal pathology are vastly different between LoF and GoF ClC-7 mutations. Loss of ClC-7 activity results in a decreased degradative capacity of lysosomes, as shown *in vivo* in renal proximal tubules (66). This effect may be due to the marked decrease in the lysosomal concentration of Cl^−^ (5, 67, 68) which is no longer accumulated by Cl^−^/H^+^-exchange. Indeed, several lysosomal enzymes depend on high Cl^−^ concentrations for activity (3, 4, 69). Lysosomal storage is mainly observed in nondividing neuronal cells (17) and in cells requiring high levels of protein degradation, such as those of renal proximal tubules (66). Lysosomal compartments in *Clcn7*^−/−^ mice may sometimes appear enlarged owing to accumulation of storage material. These cells, however, do not form the giant vacuoles generated by Cl^−^ accumulation seen in hyperactive ClC-7 mutants. In contrast to a rather slow accumulation of storage material with the loss of ClC-7, the lysosomal accumulation of Cl^−^ by hyperactive ClC-7 variants is faster and affects a broader range of tissues. Both the loss and gain of ClC-7 function can cause pigmentation defects, but the mechanisms underlying this pathology are currently unclear.

The mechanisms by which ADO-associated *CLCN7* variants with accelerated gating cause osteopetrosis also remain unclear. None of these variants were reported to markedly shift the voltage dependence to less positive potentials. As discussed above, many ADO-associated alterations displayed faster voltage-dependent activation (21). Hypothetically, this could lead to a deleterious GoF by facilitating lysosomal Ca^2+^ release, which would be very different from the GoF of HOD-causing mutations and therefore may result in different pathologies. This however, is purely speculative. One may even argue that the faster deactivation of ADO-associated variants may represent a LoF, since currents may return to baseline levels faster following depolarizing voltage transients. More work is needed to understand the pathogenic mechanisms, which may be heterogeneous, of ADO-associated *CLCN7* variants.

## Conclusions

Identification of new patients with *CLCN7*-related HOD syndrome, with one carrying a novel mutation, showed that both disease-associated mutations affect the inhibition of ClC-7 by PI(3,5)P_2_ and shift its voltage-dependent gating to more physiological lysosomal voltages. The shifted voltage-dependence is also partially conferred to WT subunits in WT/mutant ClC-7 heteromers, thereby amplifying the dominant effect of the mutated allele. Increased ClC-7 transport activity leads to the generation of large lysosome-like vacuoles which are also observed *in vivo*. *CLCN7* variants lead to a range of different lysosomal pathologies. While significant progress has been made in understanding the mechanisms underlying pathology caused by clear GoF or LoF mutations, the mechanism by which gating-accelerating mutations occurring in dominant osteopetrosis cause pathology remains unclear.

## Experimental procedures

### Subjects

The study was approved by the Ethics Committee of the Hamburg Medical Chamber. Clinical data and biological material were collected, stored, and used according to procedures in accordance with the ethical standards of the declaration of Helsinki protocols, with signed informed consents from the participating families. Photographs of the patient 1 in Figure 1 are shown with explicit permission.

### Cell culture

Human fibroblasts and *TMEM206*^−/−^ (28) HeLa cells were maintained in Dulbecco’s modified Eagles medium (Cat. P04-03550, PAN-Biotech) supplemented with 10% fetal bovine serum (Cat. P40-37500, PAN-Biotech) and 1% penicillin/streptomycin (Cat. PP06-07100, PAN-Biotech), henceforth referred to as “complete medium.” Fibroblasts were used up to a maximum passage of 15. U2OS cells constitutively expressing C-terminally enhanced GFP-tagged lysosomal marker protein LAMP1 (35) were maintained in McCoy’s 5A Medium (Cat. P04-05500, PAN-Biotech) supplemented with 10% fetal bovine serum (PAN-Biotech) and 1% penicillin/streptomycin (PAN-Biotech). All cell lines were cultured at 37 °C and 5% CO_2_.

### Electrophysiology

Plasma membrane currents were measured using a plasma membrane-targeted human ClC-7 variant, with mutated lysosomal-targeting dileucine motifs (21) inserted in a pcDNA3.1 vector (Invitrogen) that was coexpressed with human OSTM1-GFP (in a pEGFP_N1 backbone, Clontech) at 1:1 ratio of *CLCN7*:*OSTM1*. When three plasmids were coexpressed (Fig. 2, *C*–*F*), they were also mixed in equal amounts. Point mutations were introduced by site-directed mutagenesis using QuikChange (Agilent Technologies), and validated by Sanger sequencing. *TMEM206*^−/−^ HeLa cells were transfected with 1.6 μg DNA per well at 5:2 reagent:DNA ratio using Lipofectamine 2000 (Invitrogen) in a 12-well culture plate format. Cells were replated on uncoated glass coverslips on the day of recording, with currents measured 18−24 h after transfection.

Whole-cell patch-clamp experiments were performed using an EPC-10 USB patch-clamp amplifier and PATCHMASTER software (HEKA Elektronik, https://heka.com/), with a sampling rate of 20 kHz and a low-pass filter at 2 kHz. Patch pipettes had a resistance of 3 to 6 MΩ. The series resistance did not exceed 10 MΩ and was compensated for by at least 30%.

The voltage step protocol consisted of 2-s voltage steps from a holding potential of −30 mV starting from −100 to +120 mV in 20-mV increments, followed by a 500-ms step at −80 mV to assess tail currents.

The patch pipette solution contained the following (in mM): 140 CsCl, 5 EGTA, 1 MgCl_2_, 10 Hepes, with the pH adjusted to 7.2 with CsOH (275 mOsm/kg). Standard bath solution contained the following (in mM): 150 NaCl, 6 KCl, 1 MgCl_2_, 1.5 CaCl_2_, 10 D-glucose, 10 Hepes, (pH adjusted to 7.5 using NaOH) (320 mOsm/kg).

The phosphoinositides 08:0 PI(3,5)P_2_ (Сat. 850184) and 08:0 PI(3)P (Сat. 850187, Avanti Polar Lipids) were dissolved in water to a concentration of 2 mM, stored in aliquots at −70 °C, and diluted to 50 μM in the pipette solution on the day of recording.

Exponential rate constants were obtained by fitting a trace with single exponential function: $a\times e^{-x/\tau }+b$. For activation kinetics, the whole 2 s trace obtained at +120 mV was taken for analysis.

Data were analyzed using the SciPy 1.5.2 library for Python 3.8 programming language (Python Software Foundation).

### Immunocytochemistry

A total of 1×10^5^ cells were seeded on four 14 mm glass coverslips (1.5H) (Langenbrinck GmbH) per well in 6-well cell culture plates (Sarstedt). Twenty hours later, replicates of each genetic condition were treated with either 100 nM Apilimod (Cat. SML2974, Sigma-Aldrich) or 1 μM YM-201636 (Cat. HY-13228, MedChemExpress) in complete medium, or simply by a change of complete medium as a control at 37 °C and 5% CO_2_ for 3 h. Cells were then washed twice in PBS and fixed with 4% paraformaldehyde in PBS at room temperature for 15 min. Cells were washed twice with PBS and quenched with 25 mM glycine (Carl Roth) in PBS at room temperature for 5 min. Thereafter, the cells were washed thrice in 0.05% saponin (Cat. S7900, Merck)-PBS and permeabilized in PBS containing 2% sterile-filtered goat serum (NGS) (Cat. P30-1002, PAN-Biotech), 3% bovine serum albumin (BSA) (Cat. 8076.3, Carl Roth) and 0.1% saponin at room temperature for 60 min. Cells were then incubated overnight at 4 °C in primary antibody solution containing 3% BSA, 0.05% saponin, and 0.5 μg/ml monoclonal mouse antibody targeting human LAMP1 (H4A3) (Cat. MABC1108, Abcam). Cells were washed thrice the next day in 0.05% saponin-PBS and transferred to secondary antibody solution containing 3% BSA, 0.05% saponin and 4 μg/ml polyclonal goat antibody coupled to Alexa Fluor 555 (Cat. A-21424, Invitrogen), and incubated at room temperature for 60 min in the dark. The cells were washed once and nuclei were stained using 1 μg/ml DAPI (Cat. MBD0015, Merck) in 0.05% saponin–PBS at room temperature for 15 min. The coverslips were washed and mounted on Superfrost microscope glass slides (Epredia) using Fluoromount-G (SouthernBiotech) and allowed to dry. Images were acquired at room temperature under a confocal microscope using a 63X NA 1.4 oil-immersion lens (LSM880, Zeiss).

### Live cell imaging

U2OS cells were seeded on 35-mm high glass bottom (1.5H) μ-Dishes (ibidi) at a density of 2×10^5^ cells per dish 48 h before imaging. The cells were transfected 24 h before imaging with expression plasmids carrying fusion constructs of the respective human *CLCN7* variant with C-terminally RFP-tagged human *OSTM1*, separated by a P2A self-cleaving peptide sequence. Cells expressing detectable lysosomal RFP signal were deemed to sufficiently express ClC-7/OSTM1 complexes following the rationale that ClC-7 is only functional when in complex with OSTM1, the latter of which is not trafficked to the lysosome without the former. On the day of imaging, cells were washed twice in live imaging buffer containing (in mM): 135 NaCl, 5 KCl, 1 CaCl_2_, 1 MgSO_4_, 10 D-glucose, and 25 Hepes (pH adjusted to 7.4 using 1M NaOH). Nuclei were stained with 1 μg/ml Hoechst 33342 (Cat. ICT-639, Biomol) diluted in live imaging buffer for 10 min at 37 °C and 5% CO_2_. Cells were then washed once in live imaging buffer and imaged at room temperature under a confocal microscope using a 63× NA 1.4 oil-immersion lens (LSM880, Zeiss).

### Lysosomal pH measurement

Fibroblasts were seeded on glass-bottom (1.5H) 35-mm live imaging dishes (MatTek) 48 h before imaging at a density of 1×10^5^ cells per dish. The cells were incubated overnight before image acquisition with 0.5 mg/ml low-molecular weight OG 488-coupled Dextran (10 kDa) (Cat. D7170, Thermo Fisher Scientific) in growth medium. On the day of imaging, cells were washed twice and incubated in complete growth medium for 2 h at 37 °C and 5% CO_2_ in order to chase the OG 488 (OG488)-coupled Dextran into lysosomes.

Prior to imaging, cells were washed and imaged in live imaging buffer containing (in mM): 135 NaCl, 5 KCl, 1 CaCl_2_, 1 MgSO_4_, 10 D-glucose and 25 Hepes (pH adjusted to 7.4 using 1M NaOH). Each genetic condition was imaged in technical duplicate within each independent experiment. The pH was calibrated for each condition and each duplicate using clamping solutions for pH 7.0, 6.0, 5.5, 5.0, 4.5, and 4.0, containing (in mM): 135 KCl, 1 CaCl_2_, 1 MgSO_4_, 10 D-glucose and either 25 Hepes (pH 7.0), 25 Mes (pH 6.0 and 5.5) or 25 citrate (pH 5.0, 4.5 and 4.0). In addition, clamping solutions contained ionophores for K^+^ (10 μM nigericin) (Cat. N7143, Merck) and Na^+^ (10 μM monensin) (Cat. M5273, Merck) to equilibrate vesicular and applied extracellular ion concentrations for calibration post measurement. Cells were equilibrated for 5 min at room temperature before image acquisition.

Images were acquired using a Nikon Ti Eclipse widefield epifluorescence-TIRF microscope, Prime95B camera and a PE4000 CoolLed fluorescence lamp with two excitation filters: 436_20 (Zeiss 0440586) and 480_40 (Zeiss 0440583). Images were analyzed using a custom-written script in Python 3.8 programming language (Python Software Foundation). In brief, a triangle thresholding procedure was applied to background-subtracted images obtained at 435 nm (pH-insensitive wavelength) to identify regions of interest (ROI), discarding ROIs smaller than 4 pixels that likely represent noise outside of cells. The fluorescence intensity ratio at wavelengths 480:435 was calculated, and ROIs with fluorescence ratios above the 99th and below first percentile were discarded. Images with fewer than 200 ROIs, *i.e.* lysosomes, were excluded from analysis as median values from such images may be unreliable. The same procedure was performed on the images used for calibration, and ratio values were fitted with a linear function. Each technical duplicate was calibrated in the first two experiments. However, since the curves obtained for different dishes were almost indistinguishable from one another, only one dish was used for calibration in subsequent experiments. The parameters obtained from the fit were used to calculate the lysosomal pH. Median lysosomal pH was calculated for each image, then for each dish (n = 2 technical replicates per experiment). If at least for one cell line, two technical replicates yielded pH values different by >0.4 units, the whole replicate was discarded (2/9). Mean lysosomal pH between two technical replicates for each biological replicate was compared between the four cell lines using the Mann–Whitney U test with false discovery rate corrected using the Benjamini–Hochberg procedure.

Interpretation of lysosomal pH measurements using OG488-Dextran require caution. Regardless of the ratiometric nature of OG488, suboptimal loading of lysosomes in *CLCN7*^Y715C/+^ fibroblasts could lead to a higher fluorescence ratio. This is due to a larger contribution of background fluorescence in the pH-sensitive channel. Proportionally larger value in the numerator may lead to overestimation of the pH value. To mitigate such bias, poorly loaded vesicles were excluded by the thresholding procedure. This in turn results in an opposing bias favoring lower pH values. With this in mind, the pH value obtained from *CLCN7*^Y715C/+^ fibroblasts may reflect a specific subpopulation of lysosomes or lysosome-derived compartments. However, a similar tendency toward hyperacidification is also observed in *CLCN7*^K285T/+^ lysosomes with almost normal size and morphology that accumulated OG488-Dextran similar to the controls. This and a recent report of the strict dependency of apilimod-induced lysosomal hyperacidification on the activity of ClC-7 (24) lend credence to the comparison of pH values between normal lysosomes and organelles with drastically altered morphology.

### LysoTracker Red staining

In addition, 1×10^4^ cells were seeded on glass-bottom (1.5H) 35 mm live imaging dishes (MatTek) 18–20 h prior to image acquisition. The following day, cells were incubated with 75 nM LysoTracker Red DND-99 (Cat. L7528, Thermo Fisher Scientific) in complete medium at 37 °C and 5% CO_2_ for 2 h. The cells were thereafter washed twice in live imaging buffer containing in mM: 135 NaCl, 5 KCl, 1 CaCl_2_, 1 MgSO_4_, 10 D-glucose, and 25 Hepes (pH adjusted to 7.4 using 1M NaOH). Nuclei were stained using 1 μg/ml Hoechst 33342 (Biomol) diluted in live imaging buffer, allowing permeation for 5 min at 37 °C and 5% CO_2_. Cells were then washed once in live imaging buffer and imaged at room temperature under a confocal microscope using a 63× NA 1.4 oil-immersion lens (LSM880, Zeiss).

## Data availability

Materials and additional data are available from the authors upon reasonable request.

## Supporting information

This article contains supporting information (71, 72, 73, 74, 75, 76, 77, 78, 70).

## Conflict of interest

The authors declare that they have no conflicts of interest with the contents of this article.
