# Supplementary material for: Gain-of-function variants in CLCN7 cause hypopigmentation and lysosomal storage disease
Source: J Biol Chem. 2024 Jun 3;300(7):107437. doi: 10.1016/j.jbc.2024.107437 (PMC11261146; doi:10.1016/j.jbc.2024.107437)
Supplement: Supporting Information [file mmc1.pdf]

Supporting Information for "Gain-of-function variants in *CLCN7* cause hypopigmentation and lysosomal storage disease"

Table S1. Studies of lysosomal enzymes and biomarkers in two patients with HOD-associated heterozygosity for *CLCN7* variants.

As the laboratory tests for patient 2 were carried more than 25 years ago, not all reference values from that time are still available (*n/a*). Therefore, the respective interpretation of the results is indicated by colors for normal, borderline, and abnormal.

Parameter	Patient 1	Reference range	Patient 2	Reference range	Units
**Serum**					
total hexosaminidase			3164	*n/a*	nmol/hr/ml
β-hexosaminidase A			151	*n/a*	nmol/hr/ml
β-galactosidase			72	*n/a*	nmol/hr/ml
α-mannosidase			normal	*n/a*	
α-fucosidase			normal	*n/a*	
α-galactosidase			normal	*n/a*	
chitotriosidase [1])	18408	<150			nmol/hr/ml
lyso-SM-509 [1])	normal	normal			
oxysterols [1])	0.038	<0.05			ng/µl
**Urine**					
glucosaminoglycans	2…129	<34			mg/mmol$_{crea}$
oligosaccharides [2])	abnormal		normal		
neuraminic acid (bound)	181.5	19.8–48.6	57	39.0–116.8	nmol/µmol$_{crea}$
neuraminic acid (free)	71	25.5–52.7	63.6	41.0–99.8	nmol/µmol$_{crea}$
**Dried blood spots**					
total hexosaminidase	3.69	3-6			nmol/spot/21hrs
β-hexosaminidase A	0.9	0.6–2.4			nmol/spot/21hrs
β-galactosidase	0.65	0.5–3.2			nmol/spot/21hrs
α-glucosidase (with acarbose)	1.49	0.9–7.2			nmol/spot/21hrs
α-mannosidase	0.68	0.3–1.3			nmol/spot/21hrs
β-mannosidase	2.88	0.92–2.89			nmol/spot/21hrs
α-fucosidose	1.13	0.2–2.1			nmol/spot/21hrs
β-glucosidase	381	200–2000			pmol/spot/20hrs
acid sphingomyelinase	730	200–3500			pmol/spot/20hrs
α-galactosidase	148	160–2000			pmol/spot/20hrs
β-galactocerebrosidase	91	32–500			pmol/spot/20hrs
α-iduronidase	449	200–2614			pmol/spot/20hrs
iduronat-2-sulfatase	12.1	6–32			µmol/l/hr
N-acetylglucosaminidase	3.21	0.74–5.2			µmol/l/hr
galactosamin-6-sulfat sulfatase	1.39	0.8–4.4			µmol/l/hr
arylsulfatase B	16.36	10–110			µmol/l/hr
β-glucoronidase	21.62	15–52			µmol/l/hr
β-galactosidase	4.08	6–23			µmol/l/hr
total hexosaminidase	4.83	3–6			µmol/l/hr
β-hexosaminidase A	1.44	0.6–2.4			µmol/l/hr
palmitoylproteinthioesterase	0.76	0.25–2.5			µmol/l/hr
tripeptidylpeptidase	0.4	0.1–1.2			µmol/l/hr
acid lipase	1.08	0.2–2.0			µmol/l/hr
**Leukocytes**					
arylsulfatase A	12.35	7–33			nmol/hr/mg$_{prot}$
heparan-N-sulfatase	3.47	1.5–20			nmol/21hrs/mg$_{prot}$
β-galactosidase	1.31	0.2–4			nmol/min/mg$_{prot}$
β-glucocerebrosidase [3])	2.14	11.9–77.0			nmol/17hrs/mg$_{prot}$
acid lipase			normal	*n/a*	

**Fibroblasts**					
LAMP-1 [4])			~60	100	% of normal
LAMP-2 [4])			~60	100	% of normal
neuraminidase [4])<br>age of culture 6 d<br>age of culture 19 d<br>age of culture 21 d [5])			 45<br>70<br>50	 100<br>100<br>100	 % of normal<br>% of normal<br>% of normal
β-hexosaminidase A [4])<br>age of culture 6 d<br>age of culture 19 d<br>age of culture 21 d			 100<br>180<br>205	 100<br>100<br>100	 % of normal<br>% of normal<br>% of normal
neuraminidase [6])			0.8	5–15	nmol/hr/mg_{prot}
neuraminidase [7])			0.10	3–5	mU/mg_{prot}
β-galactosidase [7])			14.0	*n/a*	mU/mg_{prot}
β-hexosaminidase A [7])			80	*n/a*	mU/mg_{prot}
neuraminidase [3]), [9])	0.6	0.55–3.1	0.115 [8])	1.29–2.56	mU/mg_{prot}
β-galactosidase [3]), [9])	24.0	4.60–28.4	15.5	9.16–27.28	mU/mg_{prot}
neuraminidase [3])	46	42–237			% of normal
β-galactosidase [3])	237	45–279			% of normal
methylamine incorporation [9]), [10])			1.83	0.16–0.52	kBq/mg_{prot}
neuraminic acid (bound) [3]), [9])	6.88	4.88–15.6	19.87	0.1–4.4	nmol/mg_{prot}
neuraminic acid (free) [3]), [9])	4.67	1.12–13.9	2.02	2.2–14.9	nmol/mg_{prot}

[1]) performed by pediatric metabolic laboratory Münster
[2]) pattern on thin layer chromatography
[3]) performed by pediatric metabolic laboratory Heidelberg
[4]) kindly performed by Kurt von Figura, Göttingen
[5]) an identical reduction of enzyme activity was found irrespective of a fluorometric assay (with 4-methylumbiliferone as a substrate) or a colorimetric assay (with fetuin as a substrate) was used.
[6]) kindly performed by Klaus Harzer, Tübingen
[7]) kindly performed by Hans Kresse†, Münster
[8]) with normal K_m, reference range from (74)
[9]) kindly performed by Michael Cantz, Heidelberg
[10]) (73)

Supplementary Figures

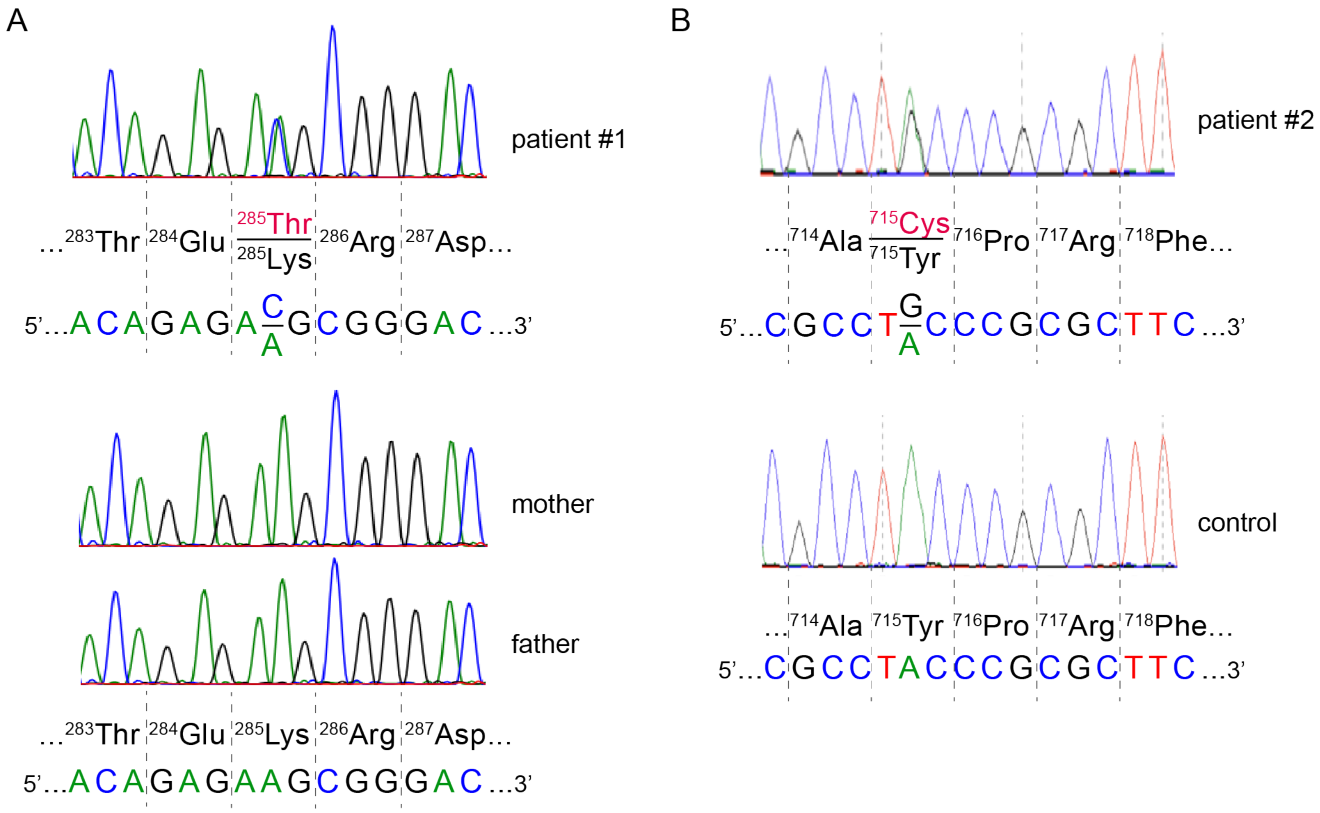

Figure S1. Sanger sequencing to verify the heterozygous mutations of the *CLCN7* gene in patient #1 (A) and patient #2 (B). **A**, Sanger sequencing traces of the patient #1 (above) and his parents (below) reveal *de novo* p.Lys285Thr variant. **B**, Sanger sequencing traces of the patient #2 (above) and an unaffected control (below) show the previously published p.Tyr715Cys variant.

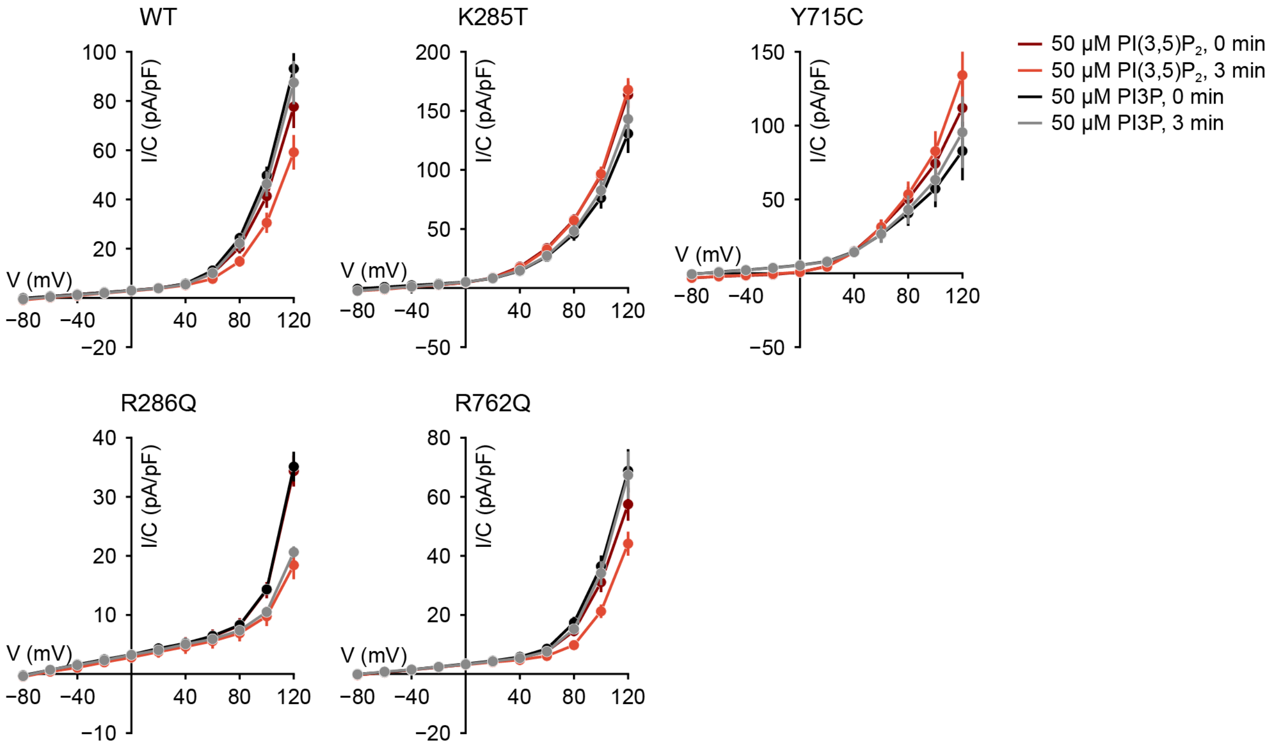

Figure S2. Current-voltage relationship of the currents presented in Figure 3B–E. Error bars represent standard error of the mean (SEM).

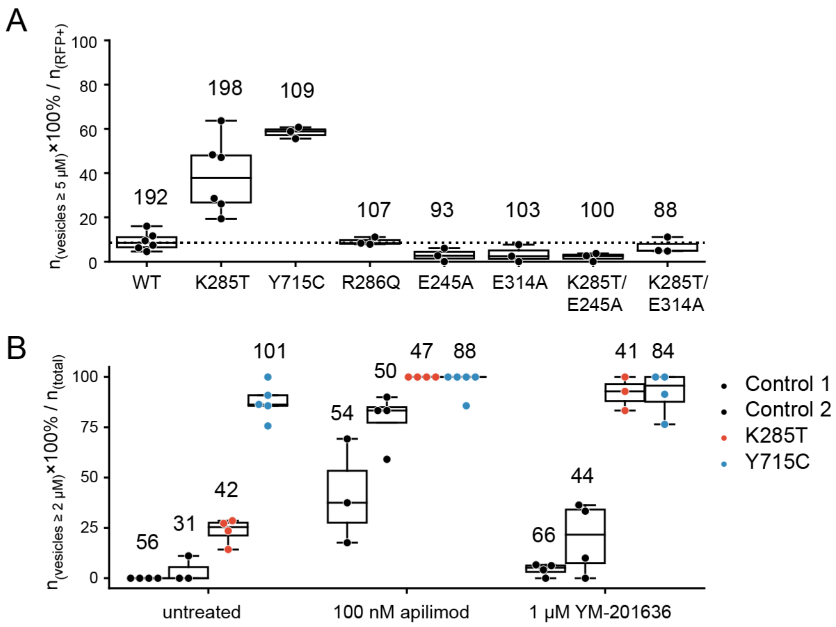

Figure S3. Quantification of microscopic images presented in Figure 4 (A) and 5A (B). **A**, Percentage of transfected (OSTM1-RFP-positive) cells that have at least one vesicle at least 5 µm in diameter. Each data point represents one independent experiment (n=3–6) with 5–11 fields of view per dish. **B**, Percentage of all fibroblasts that carry at least 5 LAMP1-positive vesicles that are over 2 µm in diameter. Each data point represents one cover-slip (n=3–6) with 3–11 fields of view. Boxes indicate the median and quartiles of the distribution. Numbers above the boxes represent the total number of cells in each condition.

Supplemental Note

Implications of extreme voltage dependence of ClC-6 and possible role of small CLC currents at less negative luminal voltages

Unlike ClC-3, -4, -5 and -7, which activate at around −20 to −30 mV (lumen-negative), ClC-6 needs V_{lum} more negative than < −100 to −120 mV for steep activation. We deem it highly unlikely that this value reflects physiological, steady state values of V_{lum}. A probably rather remote possibility would be that voltages < −100 mV may be achieved transiently. Based on their lysosomal and cytosolic concentrations, passive fluxes of Na^+, K^+, and Cl^- cannot generate such negative voltages. We propose that such voltages may be reached transiently upon the sudden release of Ca^{2+} from the lysosomes. A lysosomal $[Ca^{2+}]_{lum} \approx 500$ µM (71, 75, 76) and cytosolic $[Ca^{2+}]_{lum} \approx 100$ nM, compute the Ca^{2+} equilibrium potential to be $V_{lum} \approx -110$ mV, approaching the threshold of plasma membrane-expressed ClC-6 (54). By shunting the Ca^{2+}-current induced voltage change, ClC-6 might increase net Ca^{2+}-efflux. This consideration is relevant for WT ClC-6 and its potential LoF mutants in Kufs disease (54, 77), but not for its activating mutation in severe neurological disease (39). Here, mutations shift the activation threshold to between −20 to −30 mV.

On the other hand, WT ClC-6 also yields small currents at −20 to −30 mV (61). These currents are barely above background, but have been supported by mutagenesis studies and may be physiologically relevant. ClC-7 may also yield small currents in this voltage range, as it seems unlikely that it is completely inactivated by the combination of the acidic luminal pH and $PI(3,5)P_2$ in its native environment. At moderately cytosolic-positive or -negative voltages (up to ±20 mV), plasma membrane expressed ClC-7, which is not subject to inhibition by pH or $PI(3,5)P_2$, does not give currents that are distinguishable from background in the whole-cell configuration.

May small CLC currents at voltages where CLCs are mostly inactive have physiological effects? We estimated whether sub-background currents may still be sufficient for Cl^- accumulation in the lysosome. A HeLa cell of 20 pF has a surface area A = 20 pF / 0.01 pF/µm^2 = 2000 µm^2 (72), while a lysosome is ~5500 times smaller, with a typical volume of 0.021 µm^3 (i.e. A = 0.37 µm^2) (70). Along the endolysosomal pathway, luminal Cl^- concentration rises from ~20 mM (78) to ~120 mM (67), that is by 100 mM, or 100×10^{-3} mol/l $\times 0.021 \times 10^{-15}$ l $\times N_A \times$ mol^{-1} = 1.3×10^6 Cl^- ions in one lysosome. If complete lysosomal maturation takes one hour, 1.3×10^6 ions / 3600 s = 351 Cl^- ions have to be transported through the lysosomal membrane per second. Taking into account the exchange ratio of 1:2, i.e. Cl^- accounts for 2/3 of the transported elementary charges, ~530 charges have to be transported per second per lysosome, or $530 \times 1.6 \times 10^{-19}$ C/s = 8.5×10^{-17} A. If the density of the overexpressed transporter on the plasma membrane is assumed to be the same as the density of ClC-7 in native lysosomes, this would correspond to a whole-cell current of 8.5×10^{-17} A $\times 5500 = 4.7 \times 10^{-13}$ A, or ~0.5 pA. At 0 mV, the background conductance in our heterologous expression system is ~50 pA, 100-fold higher than the potentially physiologically relevant current amplitude of ~0.5 pA. Even if overexpression yields a transporter density 10-fold higher than the native levels, the expected physiological current density would still be 10-fold lower than the observed background. Furthermore, a recent report suggests that ClC-7 is paradoxically activated by lowering extracellular/luminal Cl^- concentration even at acidic pH (11).

Thus, we cannot exclude that the small chloride currents masked by background currents under our experimental conditions may play a physiological role – a consideration of particular relevance for WT ClC-6 and ClC-7 in their native environments (i.e. largely inhibited by $PI(3,5)P_2$ and luminal acidic pH for ClC-7). However, the concordant voltage-dependence of most endo-lysosomal CLCs and many vesicular cation channels, as well as the pathological effects of mutations shifting the voltage-dependence, strongly suggests their voltage-dependent activation at luminal negative potentials plays a pivotal biological role.
